# Impacts of rainfall shocks on out-migration are moderated more by per capita income than by agricultural output in Türkiye

**DOI:** 10.1007/s11111-023-00423-6

**Published:** 2023-06-20

**Authors:** Nathan Delacrétaz, Bruno Lanz, Amir H. Delju, Etienne Piguet, Martine Rebetez

**Affiliations:** 1 University of Neuchâtel, Neuchâtel, Switzerland; 2 ETH Zürich, Zürich, Switzerland; 3 Massachusetts Institute of Technology, Cambridge, USA; 4 Rue A.-L. Breguet 2, CH-2000 Neuchâtel, Switzerland; 5 World Meteorological Organization (WMO), Geneva, Switzerland; 6 WSL Swiss Federal Institute for Forest, Snow and Landscape Research, Zürich, Switzerland

**Keywords:** Out-migration, Climate change, Rainfall, Urbanization, Per capita income, Agriculture, Conflicts

## Abstract

Rural populations are particularly exposed to increasing weather variability, notably through agriculture. In this paper, we exploit longitudinal data for Turkish provinces from 2008 to 2018 together with precipitation records over more than 30 years to quantify how variability in a standardized precipitation index (SPI) affects out-migration as an adaptation mechanism. Doing so, we document the role of three potential causal channels: per capita income, agricultural output, and local conflicts. Our results show that negative SPI shocks (droughts) are associated with higher out-migration in rural provinces. A mediated-moderator approach further suggests that changes in per capita income account for more than one quarter of the direct effect of droughts on out-migration, whereas agricultural output is only relevant for provinces in the upper quartile of crop production. Finally, we find evidence that local conflict fatalities increase with drought and trigger out-migration, although this channel is distinct from the direct effect of SPI shocks on out-migration.

## Introduction

Ongoing changes in the climate system are responsible for an increased frequency of extreme weather events (IPCC, 2021). Because human societies fundamentally rely on climate to sustain themselves, formulating adaptation policies requires understanding how local shocks affect population dynamics. In particular, results from interdisciplinary research at the farm level suggest that extreme weather events are a key detrimental determinant of agricultural yields (e.g., Schlenker & Roberts, 2009; Burke & Lobell, 2010).^1^ In turn, societies with a predominantly rural population who rely on agriculture for subsistence and income are more exposed to increasing weather variability, and understanding adaptation mechanisms in these regions is highly policy relevant.

In this paper, we focus on out-migration as an adaptation margin (Boas & Farbotko, 2019; Borderon et al., 2019; Hoffmann et al., 2020; Call & Gray, 2020) and provide novel evidence on how random deviations from long-run precipitation patterns act as a push factor in individual migration decisions. Importantly, migration patterns are known to differ across a rural to urban dimension (see Barrios et al., 2006), and we quantify how the impact of rainfall shocks differs among rural, transitional, and urban regions. We refer to such local conditions, encompassing economic and social factors, as having a *moderating* role in the relationship between weather realizations and out-migration decisions.

In addition, we document the relevance of alternative causal channels through which random rainfall shocks affect out-migration.^2^ We refer to the factors that account for the relation between rainfall shocks and out-migration as *mediating* variables (or simply *mediators*). In this paper, we focus on the role of three mediating variables. The first is per capita GDP (Beine & Parsons, 2015; Mastrorillo et al., 2016), which captures economy-wide impacts associated with local climate-induced shocks. For example, the impact of rainfall shocks may ripple through local economic activities, not only agriculture, so that economy-wide impacts ultimately affect the broader populations living in rural regions. The second is agricultural GDP per capita (Feng et al., 2010; Cai et al., 2016), which also builds on empirical evidence at the farm level cited above. However, we note that poverty and subsistence restrictions can also imply a reduction in migration (a poverty trap, see Cattaneo & Peri, 2016). Lastly, we consider the role of conflicts as a mediating variable, as proposed by a growing literature (e.g., Burke et al., 2009; Kelley et al., 2015; Abel et al., 2019). Based on data from the Uppsala Conflict Data Program, reporting conflict-related fatalities at a highly disaggregate level, we document how rainfall shocks affect local conflicts, which in turn may affect the extent of migration out of a given province.

Our empirical approach leverages longitudinal data for 71 Turkish provinces from 2008 to 2018. Studying these data is important for at least two reasons. First, while a large strand of research on climate-related migration is conducted in low-income countries, research on middle-income countries remains scarce (see Cattaneo et al., 2019; Hoffmann et al., 2020, for a discussion). In Türkiye, we observe almost fifty provinces that are predominantly rural, although these are surrounded by either transitional or urban regions. This setting allows us to contribute to an understanding of how rural communities adapt to changes in weather shocks in a context of urbanization and structural change. Second, the Turkish Statistical Institute (TSI) provides high-quality provincial-level data, including out-migration, GDP per capita, and agricultural GDP per capita, as well as a host of other socio-demographic characteristics for each province (see also Delju et al., 2019). Similarly, the Turkish State Meteorological Service (TSMS) offers long-term precipitation records, with station-level measurement available for more than 30 years. We use these data to construct a set of standardized precipitation indices (SPI), enabling us to characterize the extent to which yearly rainfall deviates from a long-run local distribution of precipitation.^3^

Using an SPI allows us to control for differences in climatic conditions across provinces and estimate the direct effect of random rainfall shocks measured relative to a long-run distribution of rainfall for each province. In addition, we exploit the longitudinal dimension of the data to introduce fixed effects in the analysis. More specifically, we use province-level fixed effects to control for any time-invariant provincial characteristics that could affect out-migration. This would, for example, include the existence of urban agglomerations in neighboring districts or the fact that rural regions tend to experience higher out-migration on average (we come back to this below). Our analysis also controls for year fixed effects to factor out the passage of time, capturing any temporal trends in rural to urban migration (see Auffhammer & Vincent, 2012). Taken together, our empirical strategy allows us to isolate the direct impact of random deviations from the local regime of rainfall measured over the previous 30 years and quantify how these shocks act as a push factor in decisions to migrate out of each respective provinces.^4^

Building on this baseline specification, which is a well-established workhorse in the empirical literature, our contribution is twofold. First, we employ the multi-criteria analysis of Oğdül (2010) to classify each province as predominantly rural, transitional, or urban. We then use this classification as a moderating factor to estimate the impact of SPI shocks across provinces of different types.^5^ Second, we discriminate across three potential channels in how SPI shocks affect out-migration: GDP per capita, agricultural GDP per capita, and conflict fatalities. Intuitively, the resulting mediated-moderator analysis enables us to document how the channels of causality differ across provinces classified as rural, transitional, or urban.^6^

**Fig. 1:**
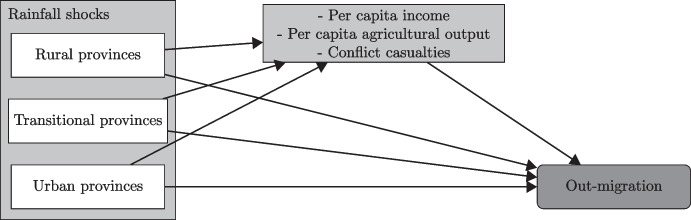
Causality paths from rainfall shocks to out-migration

The mediated-moderator approach enables us to document direct and indirect effects of randomly occurring rainfall shocks, which is novel in the context of environmental migration. Figure 1 illustrates the paths of direct and indirect causality from rainfall shocks to out-migration. The procedure for this analysis is as follows. In a first step, we identify how random shocks to long-run precipitation impact each potential mediating variable (per capita agricultural output, per capita GDP, and conflict casualties). In each case, the relationship is allowed to be different across a rural–urban typology, our moderating factor. In a second step, we estimate the direct effect of rainfall shocks on out-migration when we control for the impact of each mediating variable. This second step allows us to quantify the indirect effect of rainfall shocks on out-migration that goes through each mediator. Overall, this procedure decomposes the direct effect of rainfall shocks on out-migration across different channels.

Empirical evidence derived from our data shows that years subject to below-average SPI imply higher out-migration in rural areas. Quantitatively, a negative SPI shock of one standard deviation in the long-run distribution of rural provinces is associated with an additional 0.62 thousand emigrants on average, corresponding to a 3% increase in yearly migration out of rural provinces. We then show that this effect is mediated by GDP per capita, meaning that negative SPI shocks imply a reduction of economy-wide income in rural areas, which in turn acts as a push factor triggering out-migration. This corresponds to around 26% of the direct effect of SPI shocks on out-migration in rural province. By contrast, we do not find evidence that per capita agricultural GDP is a significant mediator at the average of the sample. In fact, our data suggest that the agricultural GDP channel is only relevant for provinces that are in the upper quartile of crop production. These results suggest that, while the agricultural channel plays a role through crop production, it is only relevant for a small share of provinces that rely heavily on these crops. Lastly, we also show that the number of conflict fatalities in rural regions tends to increase with droughts and that conflicts act as a push factor. In rural provinces, around 8% of the total effect of SPI shocks on out-migration can be attributed to conflicts. This suggests that the conflict channel operates in parallel to direct effects and depends on contextual and institutional factors (Abel et al., 2019).

These results contribute to a growing literature on the linkages between climate change and migration. While empirical evidence on this issue remains controversial (see Boas & Farbotko, 2019, for a discussion), a number of empirical studies for low-income countries provide evidence of rural–urban migration in relation to temperature shocks. This includes Marchiori et al. (2012) and Weinreb et al. (2020) for sub-Saharan Africa, Viswanathan and Kumar (2015) for India, and De Longueville et al. (2019) for Burkina Faso. Using cross-country data, Maurel and Tuccio (2016) document an impact of increasing temperature trends on urbanization, whereas Cattaneo and Peri (2016) show that poverty may prevent population movements in low-income countries, but increases them in middle-income countries.^7^ This is supported by evidence reported in Nawrotzki et al. (2016) for Mexico and Thiede et al. (2016) and Baez et al. (2017b) for South America, although further evidence on middle-income countries is needed (see Cattaneo et al., 2019, for a discussion).

We also contribute to a literature that attempts to identify the mechanisms linking climate shocks and migration. Using SPI to measure climate variability, Dallmann and Millock (2017) find that drought induces rural-rural interstate migration in India through impacts on both agricultural and total income. Similarly, Bertoli et al. (2020) report that drought increases the probability of intending to migrate, especially for low-skilled workers of rural areas, in Senegal, Niger, and Ivory Coast (for Nepal, see also Epstein et al., 2022). Another important mechanism in relation to climate shocks is conflict (Burke et al., 2015; Mach et al., 2019). Kelley et al. (2015) argue that a severe drought contributed to trigger social unrest in 2011 in Syria and being ultimately associated with mass migration, although this remains a contentious interpretation (see Selby et al., 2017; Selby, 2019). Missirian and Schlenker (2017) estimate that temperature deviations that affect agricultural yields are associated with increased asylum applications in the European Union (see also Abel et al., 2019; Cottier & Salehyan, 2021). Relative to these studies, we document the role of alternative channels in a consistent framework, showing that the mediating role differs across a rural to urban dimension.

The remaining of this paper is structured as follows. Section `Methods: Empirical strategy' provides a short discussion of the context in Türkiye and describes our empirical strategy, including our rural–urban classification and our mediated-moderator approach. Section 'Data and Results' shows a summary of our data and reports estimation results. Finally, Section `Discussion and conclusion' briefly discusses the results and concludes.

## Methods: Empirical strategy

This section first discusses the socio-demographic context of Türkyie. We then focus on our main specification to identify the impact of SPI shocks on out-migration and how this relationship is moderated by the type of provinces (rural vs. urban). Next, we present how we quantify the role of three alternative channels (or mediators) to explain the relationship between SPI shocks and out-migration: (i) GDP per capita, (ii) agricultural GDP per capita, and (iii) local conflict fatalities. Lastly, we describe how we document the robustness of our results.

### Context

This section provides a short overview of Türkiye’s socio-economic context from 2008 to 2018. During this period, Türkiye’s population grew from 70 million to 82 million, although the age structure of the population remained fairly constant, with the share of people aged 15–64 years increasing from 65 to 67%.

Ethnic composition of the country features a relatively large Kurdish population located in the eastern provinces of Türkiye. These areas have a history of tension and conflict. In line with this, we observe that violent incidents occur in eastern provinces during the 2008–2018 period, with Ankara, Hakkari, and Diyarbakir being the most affected See Appendix A. However, these conflicts do not appear to follow a specific regional trend with time.

Over the same decade, the Turkish economy also experienced fluctuations, with GDP per capita growth ranging from 0.4 to 1.8. Official unemployment increased slightly from 9.6% in 2008 to 10.9% in 2018. Importantly, the share of agriculture in GDP fell from 7.4 to 5.8%, and the share of agricultural workforce declined from 23 to 18% of the total workforce. This indicates a decrease in the role of agriculture in the economy, although the share of the workforce in this sector remains significant.

### Estimation of the main effects

Our empirical strategy is guided by the meta-analysis of Beine and Jeusette (2021) on climate change and migration. We quantify how random realization of rainfall in province *i* and year *t*, measured by variability in the SPI (denoted $\textit{SPI}_{i,t}$), affects provincial out-migration ($\textit{out-migration}_{i,t}$ in thousands of emigrants).^8^ Formally, our main regression specification is given by:

1 $$\begin{aligned} \textit{out-migration}_{i,t}=\alpha _i +\delta _t+\beta \,\cdot \, \textit{SPI}_{i,t}+\epsilon _{i,t}\,, \end{aligned}$$

where $\alpha _i$ is a set of province fixed effects controlling for any time-invariant factors that involve differences in out-migration, geological conditions, and infrastructures across provinces, and $\delta _t$ is a set of year fixed effects absorbing macro trends common across provinces, such as political conditions. $\epsilon _{i,t}$ is an error term.

The coefficient of interest is $\beta$, and the variable $\textit{SPI}_{i,t}$ is derived from station-level records of monthly rainfall from 1970 to 2020.^9^ Importantly, we do not include alternative control variables in the analysis as our objective is to measure the total effect of the SPI on out-migration (Berlemann & Steinhardt, 2017; Cattaneo & Peri, 2016; Beine & Parsons, 2015; Angrist & Pischke, 2009).^10^

To meaningfully compare rainfall shocks across provinces with potentially very different climates, our main specification employs a 12-month SPI. Intuitively, this normalizes total precipitation during 12 consecutive months in year *t* with the empirical distribution for the same 12 consecutive months observed over a period of 30 years.^11^ Therefore, observed shocks to yearly precipitation are measured relative to the long-run historical distribution of precipitation observed locally. We emphasize that the choice of a 12-month period to measure yearly precipitation allows us to focus on medium-term drought shocks (Svoboda et al., 2012).^12^

To document how the impact of the SPI on out-migration differs across rural, transitional, and urban provinces, we estimate separate coefficients $\beta$ for each type of province by defining three moderating variables: $\textit{Rural}_{i}$ equals one if the province *i* is predominantly rural, zero otherwise; $\textit{Urban}_{i}$ is one if *i* predominantly urban, zero otherwise; and $\textit{Transitional}_{i}$ equals one if *i* is neither predominantly urban nor rural, zero otherwise. We refer to these variables as moderators because they can potentially change the estimated value of $\beta$.

**Fig. 2:**
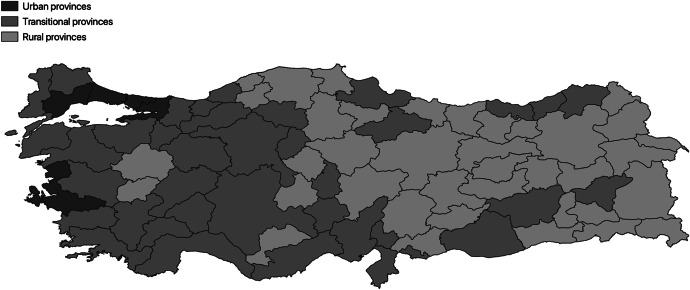
Classification of Turkish provinces (source: Oğdül, 2010)

The moderating variables are based on a detailed multi-criteria classification by Oğdül (2010), which defines a province as rural if at least 50% of its constituting districts are classified as rural, urban if 50% or more of its districts are urban, and transitional if it is neither rural nor urban. District-level classification is then based on six categories of socio-demographic characteristics: agricultural production, non-agricultural production, employment structure, demography, educational level, and trade opportunities (see Appendix C for a comprehensive list of factors). The resulting classification comprises 32 rural provinces, 34 transitional provinces, and 5 urban provinces, as illustrated in Fig. 2. In the robustness checks section below, we discuss alternative approaches to distinguish between rural and urban provinces.

We employ these data to augment the Eq. 1 as follows:

2 $$\begin{aligned} \textit{out-migration}_{i,t}=\alpha _i +\delta _t+\beta _1 \,\cdot \, \textit{SPI}_{i,t} \times \textit{Rural}_{i} + \beta _2 \,\cdot \, \textit{SPI}_{i,t} \times \textit{Transitional}_{i}+ \beta _3 \,\cdot \, \textit{SPI}_{i,t} \times \textit{Urban}_{i} +\epsilon _{i,t}\,. \end{aligned}$$

By interacting the variable $\textit{SPI}_{i,t}$ with each moderating variable, we quantify how local SPI shocks affect out-migration for each type of province.^13^ Importantly, out-migration is expected to differ in rural versus urban provinces for reasons that are not related to rainfall shocks, such as economic opportunities (Beine & Jeusette, 2021; Marchiori et al., 2012). However, we emphasize that these drivers of emigration are controlled by including province-level fixed effects ($\alpha _i$) that capture time-invariant structural characteristics of each province.

### Estimation of potential channels: mediated-moderator analysis

We now present how we quantify the mediating role of economic activities and conflicts. Specifically, we use data on per capita GDP and agricultural GDP from TSI as well as the number of conflict fatalities from the Uppsala Conflict Data Program.^14^ To quantify the relevance of each potential channel, we employ the mediated-moderator specification of Morgan-Lopez and MacKinnon (2006). This requires two steps. First, we estimate the impact of SPI shocks on each mediating variable, denoted $\textit{Y}_{i,t}$, across each type of province:

3 $$\begin{aligned} \textit{Y}_{i,t}=\eta _i + \phi _1 \, \textit{SPI}_{i,t} \times \textit{Rural}_{i} + \phi _2 \, \textit{SPI}_{i,t} \times \textit{Transitional}_{i} + \phi _3 \, \textit{SPI}_{i,t} \times \textit{Urban}_{i} +\chi _t+\varepsilon _{i,t}\,, \end{aligned}$$

where the notation follows the same logic as in Eq. 2. This equation allows us to document whether provincial-level SPI shocks have an impact on each mediator variable $\textit{Y}_{i,t}$ across our rural to urban classification, a necessary condition for mediated-moderator analysis (MacKinnon et al., 2007).

The second step of our mediated-moderator analysis quantifies the impact of SPI shocks on out-migration, akin to Eq. 2, but we also include each respective mediating variable $\textit{Y}_{i,t}$ in the regression. Formally:

4 $$\begin{aligned} \textit{out-migration}_{i,t}&=\alpha _i +\delta _t+\beta _1 \,\cdot \, \textit{SPI}_{i,t} \times \textit{Rural}_{i} + \beta _2 \,\cdot \, \textit{SPI}_{i,t} \\ &\quad\times \textit{Transitional}_{i} + \beta _3 \,\cdot \, \textit{SPI}_{i,t} \times \textit{Urban}_{i} + \psi \, \textit{Y}_{i,t} +\epsilon _{i,t}\,. \end{aligned}$$

Therefore, while the first step in Eq. 3 quantifies the impact of SPI shocks on each mediating variable, Eq. 4 controls for indirect effects linking each mediating variable and out-migration in our main specification (see also Fig. 1). Indirect effects of SPI shocks are accounted for by changes in the mediating variable (MacKinnon et al., 2007). This allows us to assess whether SPI shocks have a stand-alone effect on out-migration once we control for contemporaneous changes in the mediating variable. In particular, evidence that the effect of SPI shocks on out-migration vanishes in Eq. 4 would indicate that the mediating variable acts as a channel for the direct relationship.

### Robustness checks

We document the robustness of our results along four key dimensions: (i) the definition of SPI shocks; (ii) socio-demographic factors; (iii) our rural–urban classification of provinces; and (iv) our measure of the migration response. In the following, we briefly explain how we implement each robustness check in turn.

We start by documenting the role of alternative definitions of SPI shocks and consider the possibility of more long-term impacts based on 24-month and 36-month SPIs. This allows us to evaluate how longer deviations from the historical distribution of precipitation records affect out-migration flows. Related to that, we further test for the presence of year-on-year spillover effects by re-estimating Eq. 1 with a 1-year lag for the 12-month SPI (instead of contemporaneous impacts). Next, we focus on the effect of drought events and construct an indicator variable that counts the number of successive years with a SPI smaller or equal to −1. This identifies years in which the amount of precipitation is less than one standard deviation below the long-term average, while also taking into account the possible drought that occurred in previous years. Lastly, we control for the role of temperature by using a standardized precipitation-evapotranspiration index (SPEI). This allows us to take into account evapotranspiration, which plays an important role in agricultural yields (see Proctor et al., 2022).

Next, we study how socio-demographic factors affect the total effect of SPI shocks on out-migration and consider a specification in which we control for three key factors. The first is education (Findley, 1994; Baez et al., 2017a; Kabir et al., 2018), and we include the share of population with primary education, the share of population with higher education, and the share of young adults (aged 15 to 24 years) in the population. Second, we control for the share of men per women (the sex ratio, see Gray & Mueller, 2012; Nawrotzki et al., 2016; Debnath & Nayak, 2020). As noted in Berlemann and Steinhardt (2017) and Cattaneo and Peri (2016), however, these variables are potentially affected by SPI shocks, so these results should be interpreted with caution. Third, we control for population density and use its value for $t-1$ in order to mitigate potential endogeneity concerns associated with this variable (Burke et al., 2009; Couttenier & Soubeyran, 2014).

Turning to the role of rural provinces, we start by employing two alternative approaches to our rural–urban classification. First, we use the share of provincial population living in cities with more than 300,000 inhabitants. Second, we consider the share of provincial population working in agriculture. Therefore, we re-estimate Eq. 1 interacting the 12-month SPI with each variable to document whether $\beta$ varies continuously along with these two dimensions. Next, we consider the possibility that access to irrigation may be different in rural, transitional, and urban areas, which in turn may buffer local shocks (as in Benonnier et al., 2019). For this purpose, we employ TSI data on the share of irrigated agricultural land in each province (available for 2003) and interact them with our SPI measure.^15^ Fourth, we document the importance of crop production in rural provinces. To do so, we divide rural provinces into 3 different categories based on quartiles for 2007 crop production (in tons). Specifically, “high crop production” provinces are those above the 75th percentile, “medium crop production” are those provinces between the 75th and 25th percentiles, and “low crop production” are provinces below the 25th percentile. Based on this, we quantify how rainfall shocks differently impact GDP per capita and agricultural GDP per capita across rural districts with varying levels of crop production.

Our final robustness checks employ three alternative measures for migration. In a first step, we scale our measure of out-migration by province-level population data and re-estimate our main Eq. 2. Second, we transform the outcome variable with a natural logarithm, allowing us to estimate proportional (percentage) results across provinces. Lastly, we exploit data on net migration rates at the province level, which is defined as the difference between in-migration and out-migration, scaled by provincial population. As our study focuses on push factors, using net migration as an outcome variable can obscure some of our results by accounting for pull factors as well. Nevertheless, considering net migration provides further confidence in the validity of our main estimates.

## Data and results

This section reports our empirical results. First, we provide summary statistics for our sample. Second, we present the results from our main specification, documenting the impact of SPI shocks on out-migration across our rural–urban classification. Third, we discuss the results of our mediated-moderator analysis. Fourth, we present the results of the robustness checks.

### Descriptive statistics

Table 1 provides summary statistics for rural, transitional, and urban provinces. Out-migration tends to be larger and more volatile in provinces classified as urban, although as a percentage to total population it is larger in rural provinces (4.82%) relative to both transitional (3.85%) and urban (3.34%) provinces. On average across all provinces out-migration is 31.78 thousands of emigrants each year, or around 4% of the provincial population.

**Table 1:** Descriptive statistics across Turkish provinces

	Mean	Std. Dev	SD:mean ratio	Min	Max
*Rural provinces (N = 32)*					
Out-migration	19.07	10.41	0.55	4.09	62.21
12-month SPI	0.17	1.05	6.18	$-$2.98	2.82
GDP per capita	6.72	1.63	0.24	2.73	11.64
Agricultural GDP per capita	2.76	1.65	0.6	0.4	11.61
Conflict fatalities	0.07	0.31	4.43	0	3.77
*Transitional provinces (N = 34)*					
Out-migration	30.93	27.23	0.88	4.61	221.75
12-month SPI	0.19	1.06	5.58	$-$2.57	3.23
GDP per capita	8.99	2.67	0.3	3.31	17.59
Agricultural GDP per capita	2.84	1.36	0.48	0.4	7.61
Conflict fatalities	0.03	0.23	7.7	0	3.56
*Urban provinces (N = 5)*					
Out-migration	118.91	147.72	1.24	6.54	595.8
12-month SPI	0.2	1.12	5.6	$-$2.09	2.9
GDP per capita	14.77	3.08	0.21	8.96	20.73
Agricultural GDP per capita	0.92	0.68	0.74	0.04	2.27
Conflict fatalities	0.03	0.17	5.67	0	1.16

Data sources are TSI, TSMS, and Uppsala Conflict Data Program, from 2008 to 2018. *Out-migration* is in thousand of emigrants. *Conflict fatalities* is the number of fatalities reported for local conflicts (in hundred)

The mean and variability for the 12-month SPI are very similar across provinces, which is implied by the way it is constructed. In particular, the SPI measures deviation in rainfall relative to a province-specific distribution measured over 30 years. Crucially, however, our identification strategy takes advantage of the random timing and magnitude of shocks to our 12-month SPI. We note that, for each type of province, the average SPI is slightly higher than zero, which indicates that precipitations are on average slightly higher compared to historical records.^16^

Other variables follow an expected pattern, with GDP per capita being significantly higher in urban provinces, followed by transitional and rural provinces, whereas agricultural GDP per capita is similar among rural and transitional provinces but substantially lower in urban provinces. We also note that the number of conflict fatalities is, on average, around two times larger in rural provinces, although the maximum is relatively close for rural and transitional provinces and significantly lower in urban provinces.

### The impact of SPI shocks on out-migration in rural, transitional, and urban provinces

Table 2 reports regression results quantifying the impact of SPI shocks on out-migration (Eqs. 1 and 2). Column 1 is a simple bivariate regression of SPI on out-migration (no fixed effects). In column 2, we add province and year fixed effects to control for, respectively, all time-invariant provincial characteristics and common macro shocks. In column 3, we estimate separately impacts of SPI shocks for rural, transitional, and urban provinces (Eq. 2). In all columns, we report standard errors clustered at the province level in parentheses.

**Table 2:** Baseline results for the impact of SPI shocks on out-migration

	*Outcome: Out-migration in thousand of emigrants*		
	Bivariate	FE	FE
	(1)	(2)	(3)
SPI	$-$1.97	0.01	−
	(1.66)	(0.20)	
SPI$\times$Rural	−	−	$-$0.62**
			(0.27)
SPI$\times$Transitional	−	−	$-$0.38*
			(0.20)
SPI$\times$Urban	−	−	2.25
			(1.60)
Constant	32.05***	28.53***	28.32***
	(1.85)	(0.77)	(0.87)
Fixed effects	No	Yes	Yes
Number of observations	776	776	776
Number of provinces	71	71	71
Adjusted$R^{2}$	0.01	0.09	0.10

Results from linear regressions reported. *SPI* is the 12-month SPI per year and province. *Rural*, *transitional,* and *urban* are indicator variables for rural, transitional, and urban provinces, respectively. The period of observation is from 2008 to 2018. In columns 2 and 3, we include province and year fixed effects. In all columns, we report standard errors clustered at the province level in parentheses. *,**, and *** respectively denote statistical significance at 10%, 5%, and 1%

Results in column 1 indicate that a one standard deviation increase in SPI, which represents a year with relatively abundant precipitation, is associated with a decrease of out-migration by 1.97 thousand emigrants on average. However, this coefficient is not accurately estimated. Furthermore, the introduction of fixed effects substantially reduces the magnitude of the coefficient (see Auffhammer & Vincent, 2012, for a similar result).

More interestingly, column 3 shows that decomposing the total effect of SPI across rural, transitional, and urban provinces implies very different results. For rural provinces, there is a negative and statistically significant effect of SPI shocks in rural provinces (*p*-val. < 0.05). This indicates that a drought, which represents a negative SPI shock, is associated with an increase of out-migration in rural provinces on average. Quantitatively, a one-standard deviation *decrease* in SPI *increases* out-migration in rural provinces by 0.62 thousand emigrants on average, which is around 3% of the average annual out-migration in rural provinces.

We also estimate that droughts tend to increase out-migration in transitional provinces, as the coefficient is negative and borderline statistically insignificant (*p*-val. < 0.1). Lastly, the point estimate for urban provinces is positive and large relative to other provinces, although it does not reach statistical significance at conventional levels. One potential interpretation of this result is that urbanized provinces are less vulnerable to climate shocks than other provinces when exposed to precipitation shocks. In addition, a higher degree of diversification in economic activities may help to retain population in the presence of shocks. However, given the relatively small number of urban provinces in our sample, these estimates should be interpreted with caution.

Overall, our data suggest that a drought increases out-migration in rural provinces and that it has a smaller and less precisely estimated impact for transitional provinces, whereas the effect for urban provinces is large but statistically insignificantly different from zero. Taken together, these effects cancel out on average so that we find no direct effect of SPI shocks on migration when we do not consider the urban-rural classification of provinces (columns 1 and 2).

### Channels: GDP per capita, agricultural GDP per capita, and conflict fatalities

We now turn to our mediated-moderator analysis to discriminate across three potential channels linking SPI shocks and out-migration. In Table 3 we report the results for each mediating variable: GDP per capita in columns 1 and 2, agricultural GDP per capita in columns 3 and 4, and conflict fatalities in columns 5 and 6. Furthermore, results for the first step estimation (Eq. 3), which quantifies the impact of SPI shocks on each mediating variable, are in columns 1, 3, and 5. Results for the second step of the channel analysis (Eq. 4), where we estimate the effect of SPI shocks on out-migration controlling for the mediating variable, are in columns 2, 4, and 6. In all columns, we include province and year fixed effects and report standard errors clustered at the province level in parentheses.

**Table 3:** Analysis of channels for the impact of SPI shocks on out-migration

*Specification:*	Channel: GDP		Channel: Ag. GDP		Channel: Conflicts	
*Outcome:*	GDP p.c	Out-migration	Ag. GDP p.c	Out-migration	Conflicts	Out-migration
	(1)	(2)	(3)	(4)	(5)	(6)
SPI$\times$Rural	0.05**	$-$0.46*	$-$0.01	$-$0.65**	$-$0.02**	$-$0.57**
	(0.02)	(0.24)	(0.03)	(0.29)	(0.01)	(0.26)
SPI$\times$Transitional	$-$0.05	$-$0.53**	0.08*	$-$0.05	0.01	$-$0.41**
	(0.03)	(0.24)	(0.04)	(0.22)	(0.01)	(0.20)
SPI$\times$Urban	$-$0.33***	1.22	$-$0.04	2.10	0.01	2.22
	(0.11)	(1.31)	(0.04)	(1.62)	(0.01)	(1.58)
*Mediating variables*						
GDP per capita	-	$-$3.08**	-	-	-	-
		(1.40)				
Agricultural GDP per capita	-	-	-	$-$4.20*	-	-
				(2.27)		
Conflict fatalities	-	-	-	-	-	3.63**
						(1.77)
Constant	8.21***	53.63***	1.53***	34.73***	0.04***	28.19***
	(0.06)	(11.11)	(0.08)	(2.92)	(0.01)	(0.88)
Fixed effects	Yes	Yes	Yes	Yes	Yes	Yes
Number of observations	776	776	776	776	776	776
Number of provinces	0.77	0.12	0.68	0.15	0.05	0.11
Adjusted$R^{2}$	71	71	71	71	71	71

Results from linear regressions reported. *SPI* is the 12-month SPI per year and province. *Rural*, *transitional,* and *urban* are indicator variables for rural, transitional, and urban provinces, respectively. *GDP p.c.* is per capita GDP, *Ag. GDP p.c.* is agricultural GDP per capita, and c*onflict fatalities* is the number of fatalities in conflicts (in hundreds). The period of observation is from 2008 to 2018. In all columns, we include province and year fixed effects and report standard errors clustered at the province level in parentheses. *,**, and *** respectively denote statistical significance at 10%, 5%, and 1%

Column 1 shows that SPI shocks in rural provinces have a positive and statistically significant effect on GDP per capita (*p*-val. < 0.05). This implies that droughts (negative SPI shocks) are associated with a decrease in GDP per capita in rural provinces. Evidence for this relationship is a necessary condition for the mediated-moderator analysis (MacKinnon et al., 2007; Morgan-Lopez & MacKinnon, 2006). Furthermore, column 2 provides evidence that GDP per capita has a negative and statistically significant association with out-migration (*p*-val < 0.05). One implication is that a decrease in per capita income acts as a push factor in migration decisions. More importantly, results in column 2 suggest that the coefficient for SPI shocks in rural provinces is significantly smaller compared to the results in Table 2, column 3, and still statistically significant at 10%. Taken together, these results imply that GDP per capita mediates around 26% ($=(-0.62-(-0.46))/-0.62$) of the total impact of SPI shocks on out-migration in rural provinces.

In urban provinces, the results of column 1 suggest that SPI shocks have a negative and statistically significant impact on GDP per capita (*p*-val. < 0.01). However, in column 2, the coefficient for SPI shocks on out-migration is significantly smaller when we control for GDP per capita in our main estimation. One potential interpretation is that the impact of SPI on out-migration in urban provinces is fully driven by per capita GDP, although the coefficient for the second stage remains imprecisely estimated and statistically insignificant (notwithstanding the relatively small number of urban provinces). In transitional provinces, we do not find precise evidence that SPI shocks affect GDP per capita.

Next, results presented in column 3 suggest that SPI shocks do not significantly affect agricultural output in rural and urban provinces and have a positive but loosely statistically significant impact in transitional provinces (*p*-val < 0.1). In line with this, column 4 shows that introducing agricultural GDP per capita in our main specification does not significantly affect point estimates quantifying the effect of SPI shocks on out-migration. In other words, adding GDP per capita in Eq. 2 does not significantly change our estimated effect relative to Table 2, column 3. This suggests that agricultural GDP is not a mediating variable in the relationship between SPI shocks and out-migration in rural provinces. We come back to this result in the robustness checks and show that these are driven by the volume of crop production (see Section `Robustness checks').

Lastly, column 5 provides evidence about the relationship between SPI shocks and conflict casualties. Results for rural provinces indicate that an increase in SPI (rainy year) implies a decline in the number of fatalities (*p*-val. < 0.05), whereas there are no effects for transitional and urban provinces. Furthermore, column 6 shows that conflicts have a positive and statistically significant association with out-migration (*p*-val < 0.05). As expected, an increase in conflict fatalities acts as a push factor in migration decisions. In turn, controlling for conflicts reduces the impact of SPI shocks on out-migration in rural provinces as compared to Table 2, column 3. The share of the total effect of SPI on out-migration mediated by conflicts is around 8% ($=(-0.62-(-0.57))/-0.62$). For urban and transitional provinces, we do not find evidence that conflicts act as a mediating variable.

### Robustness checks

Table 4 reports the results of robustness checks using alternative measures of rainfall shocks in our main specification (Eq. 2). In column 1, we use a 24-month SPI, in column 2, a 36-month SPI, and in column 3, we use a 12-month SPI together with its lagged value. In column 4, we use a count for the number of successive years in which a 12-month SPI is equal or below −1. In column 5, we use a 12-month SPEI. Lastly, in column 6, we include a vector of socio-demographic control variables. In all columns, we include province and year fixed effects and report standard errors clustered at the province level in parentheses.

**Table 4:** Robustness results for the impact of rainfall shocks on out-migration

	24-month SPI	36-month SPI	Lagged SPI	Drought years	12-month SPEI	Control variables
	(1)	(2)	(3)	(4)	(5)	(6)
SPI$\times$Rural	$-$0.32	$-$0.46	$-$0.65**	-	-	$-$0.49**
	(0.29)	(0.34)	(0.29)			(0.21)
SPI$\times$Transitional	$-$0.46	$-$0.49	$-$0.41*	-	-	$-$0.03
	(0.34)	(0.53)	(0.24)			(0.19)
SPI$\times$Urban	0.12	$-$0.3	2.1	-	-	3.41
	(0.43)	(0.9)	(1.5)			(3.02)
Lagged SPI$\times$Rural	-	-	0.08	-	-	-
			(0.26)			
Lagged SPI$\times$Transitional	-	-	$-$0.23	-	-	-
			(0.17)			
Lagged SPI$\times$Urban	-	-	$-$1.39	-	-	-
			(1.48)			
Drought$\times$Rural	-	-	-	1.34*	-	-
				(0.69)		
Drought$\times$Transitional	-	-	-	1.16**	-	-
				(0.54)		
Drought$\times$Urban	-	-	-	$-$15.94	-	-
				(11.51)		
SPEI$\times$Rural	-	-	-	-	$-$0.65**	-
					(0.3)	
SPEI$\times$Transitional	-	-	-	-	$-$0.17	-
					(0.19)	
SPEI$\times$Urban	-	-	-	-	1.4	-
					(1.42)	
Constant	28.35***	28.31***	28.17***	28.60***	28.387***	21.07
	(0.92)	(0.99)	(0.93)	(0.82)	(0.88)	(20.19)
Fixed effects	Yes	Yes	Yes	Yes	Yes	Yes
Control variables	No	No	No	No	Yes	Yes
Number of observations	776	776	771	776	776	776
Adjusted$R^{2}$	0.09	0.1	0.1	0.12	0.1	0.61
Number of provinces	71	71	71	71	71	71

Results from linear regressions reported. In columns 1 and 2, the variable *SPI* is a 24-month and 36-month SPI, respectively. In column 3, we include a 12-month SPI together with its lagged value. In column 4, the variable d*rought* is the count of successive years in which the 12-month SPI is equal or below −1. In column 5, the variable *SPEI* is a 12-month SPEI. In column 6, we control for the share of population above 15 years with primary and higher education, the ratio of male per female, the share of population between 15 and 24 years and lagged population density. All columns include province and year fixed effects. Standard errors clustered at the province level reported in parentheses. *,**, and *** respectively denote statistical significance at 10%, 5%, and 1%

Estimates in columns 1 and 2 show that using a 24- and 36-month SPI implies relatively similar patterns for rural provinces compared to a 12-month SPI, although point estimates are smaller and statistically insignificant. Similarly, introducing a lagged 12-month SPI (column 3) does not affect the magnitude of contemporaneous effects, whereas the coefficients for the lagged variables are small and statistically insignificant. Overall, these results suggest that the impact of rainfall shocks on out-migration is larger in the short term. However, these effects remain persistent for at least 3 years. Column 4 suggests that an additional year of drought tends to increase out-migration in both rural and transitional districts, with a pattern that is close to our baseline specification. This suggest that a long-lasting drought has a larger effect on out-migration.

Estimates in column 5 of Table 4 show that adding temperature to our index does not significantly change our results. This is important because the potential for evapotranspiration occurring during drought periods does not significantly affect our results. Column 6 suggests that our results are robust to the addition of socio-demographic control variables, as we estimate a statistically significant and negative effect for rural provinces (*p*-val. < 0.05). This shows that our main results are robust to omitted socio-demographic factors, such as the age structure of the population.

We now turn to our second set of robustness checks and focus on rural provinces. Results are reported in Table 5. In columns 1 and 2, we interact the 12-month SPI with, respectively, the share of population living in cities of more than 300,000 inhabitants and the share of population working in agriculture. In column 3, we include an interaction between the share of irrigated land (measured in 2003) and the 12-month SPI. Columns 4 to 5 consider the impact of SPI shocks on GDP per capita and agricultural GDP per capita (Eq. 3) accounting for crop production in rural provinces.

**Table 5:** Robustness results for rural provinces

*Outcome:*	*Out-migration in thousand of emigrants*			*GDP p.c.*	*Ag. GDP p.c.*
	Urban population	Ag. labor	Irrigation	Crops production intensity	
	(1)	(2)	(3)	(4)	(5)
SPI	$-$0.36**	1.76*	-	-	-
	(0.16)	(0.91)			
﻿SPI$\times$% urban	0.60***	-	-	-	-
	(0.08)				
SPI$\times$ag. labor share	-	$-$5.58**	-	-	-
		(2.51)			
SPI$\times$Rural	-	-	$-$0.66**	-	-
			(0.29)		
SPI$\times$Transitional	-	-	$-$0.62	$-$0.05	0.08**
			(0.5)	(0.03)	(0.04)
SPI$\times$Urban	-	-	3.70*	$-$0.33***	$-$0.03
			(2.16)	(0.11)	(0.04)
SPI$\times$Rural$\times$Irrigation	-	-	0.11	-	-
			(0.17)		
SPI$\times$Transitional$\times$Irrigation	-	-	0.61	-	-
			(0.95)		
SPI$\times$Urban$\times$Irrigation	-	-	$-$2.60*	-	-
			(1.55)		
SPI$\times$Rural$\times$High crops production	-	-	-	0.07*	0.14**
				(0.03)	(0.06)
SPI$\times$Rural$\times$Medium crops production	-	-	-	0.06**	$-$0.02
				(0.03)	(0.03)
SPI$\times$Rural$\times$Low crops production	-	-	-	0.02	$-$0.10***
				(0.04)	(0.02)
Constant	28.49***	28.68***	28.32***	8.21***	1.53***
	(0.84)	(0.75)	(0.89)	(0.06)	(0.08)
Fixed effects	Yes	Yes	Yes	Yes	Yes
Number of observations	776	776	776	776	776
Number of provinces	0.11	0.1	0.1	0.77	0.68
Adjusted$R^{2}$	71	71	71	71	71

Results from linear regressions reported. In columns 1 to 3, the dependent variable is out-migration in thousand of emigrants and the dependant variable is a 12-month SPI. In columns 1 and 2, we interact a 12-month SPI with *% urban* (share of population in cities) and *Ag. labor share* (share of labor force in agriculture), respectively. In column 3, we measure the effect of interact i*rrigation* (share of irrigated hectares in 2003) for *rural*, *transitional,* and *urban* provinces. In column 4, the outcome variable is GDP per capita, and in column 5, it is agricultural GDP per capita, and we quantify how SPI shocks in rural provinces in relation to 2007 crop production (*high crop production*, *medium crop production,* and *low crop production*). In all columns, we include province and year fixed effects and report standard errors clustered at the province level in parentheses. *,**, and *** respectively denote statistical significance at 10%, 5%, and 1%

Results in column 1 suggest that provinces with a higher proportion of urban residents tend to experience greater positive impacts of SPI shocks (*p*-val. < 0.01). Similarly, column 2 shows that an increase in the share of agricultural labor implies more negative impacts associated with SPI shocks (*p*-val. < 0.05). These results are consistent with the analysis above. Column 3 suggests that larger irrigated area implies a smaller out-migration response to SPI shocks, so that irrigation acts as a buffer, although the effect is not statistically significant at conventional levels.

Estimates from column 4 of Table 5 show that SPI shocks have a positive impact on GDP per capita in rural provinces with high and medium crop production, which is consistent with results in Table 3, column 1. More interestingly, column 5 shows that the impact of SPI shocks on agricultural GDP per capita is of opposite sign for rural provinces with high and low intensity in crop production. For provinces with high crop production, the impact is positive, whereas the effect is negative in provinces with low crop production. For provinces with medium crop production, the impact of SPI shocks is small and not statistically significant. These results suggest that SPI shocks have heterogeneous impacts across rural provinces and explain the lack of evidence when considering agricultural GDP as a channel (column 3 of Table 3). Our hypothesis regarding these results is that provinces that are less dependent on crops experience less damage to their agricultural production during droughts. This can lead to a substitution effect, where goods that can be produced under high climate stress experience increased demand and/or higher prices. In turn, impacts on agricultural GDP per capita would be mitigated.

**Table 6:** Robustness results for alternative measures of migration

	% out-migration	$\ln$(out-migration)	Net-migration
	(1)	(2)	(3)
SPI$\times$Rural	$-$0.07*	$-$0.11	1.31*
	(0.03)	(0.07)	(0.71)
SPI$\times$Transitional	$-$0.01	0.01	0.89***
	(0.03)	(0.08)	(0.29)
SPI$\times$Urban	0.12***	0.31***	$-$2.04**
	(0.02)	(0.07)	(0.85)
Constant	4.00***	29.61***	$-$2.49**
	(0.07)	(0.15)	(0.95)
Fixed effects	Yes	Yes	Yes
Number of observations	776	776	776
Number of provinces	0.14	0.38	0.14
Adjusted$R^{2}$	71	71	71

Results from linear regressions reported. In column 1, the dependent variable is out-migration divided by provincial population, and in column 2, it is the natural log of out-migration. In column 3, it is net migration measured as the difference between in-migration and out-migration divided by provincial population. *SPI* is the 12-month SPI per year and province. Rural, transitional, and urban are indicator variables for rural, transitional, and urban provinces, respectively. The period of observation is from 2008 to 2018. In all columns, we include province and year fixed effects and report standard errors clustered at the province level in parentheses. *,**, and *** respectively denote statistical significance at 10%, 5%, and 1%

The last set of robustness checks focus on alternative migration measures. Results reported in Table 6 largely confirm previous findings. First, when the outcome is measured as fraction of provincial population (column 1), the coefficient for rural provinces implies that a negative one-standard deviation SPI shock increases out-migration in rural provinces by around seven percentage points (*p*-val. < 0.1).^17^ When we consider a logarithmic-transformed measure of out-migration (column 2), the impact of SPI shocks in rural provinces remains negative, although it is not statistically significantly different from zero. We note, moreover, that columns 1 and 2 suggest positive and statistically significant effects of SPI shocks on out-migration in urban districts. Finally, results for net-migration in column 6 suggest that SPI shocks have a positive impact in rural provinces (*p*-val. < 0.1). An implication is that drought years are associated with an overall decline in net migration.

## Discussion and conclusion

This study has contributed to an understanding of the relationship between variability in rainfall and migration, providing novel empirical evidence on how SPI shocks affect out-migration across Turkish provinces. We have shown that the relationship is moderated by whether a province is rural, transitional, or urban, with evidence that drought events imply increased migration out of rural provinces. We have also quantified the mediating role of per capita GDP as a channel to explain higher out-migration as a response to negative SPI shocks in rural provinces. One implication of our data is that droughts induce a decrease in per capita GDP, which in turn acts as a push factor in out-migration decisions. Evidence further suggests that agricultural GDP is also a push factor in the case of a drought, but only for rural provinces with relatively important crop production. This result complements studies by Feng et al. (2010) and Cai et al. (2016), which also emphasize the importance of shocks to agricultural output in migration decisions.

One interpretation of our results is that provinces with low level of urbanization are more exposed to climate variability, making it more likely that precipitation shocks will act as a push factor in migration decisions. This is similar to previous research that has shown that countries with a high dependence on agriculture and a lack of capacity and infrastructure to cope with climate shocks are particularly vulnerable (see Cattaneo et al., 2019). However, the mechanism linking droughts and migration in rural areas is more complex than a simple impact on the agricultural sector. One possible explanation is that price fluctuations for crops can impact the entire economy, so that for provinces with relatively large crop production where the agricultural sector makes up a larger share of the local economy, agricultural GDP is more directly affected by fluctuations in the SPI. Further research is needed to confirm this interpretation.

Furthermore, our analysis shows that conflicts also increase with droughts and play a role as a push factor in out-migration decisions, which is consistent with evidence from other contexts (Kelley et al., 2015; Missirian & Schlenker, 2017). Our data further suggest that conflict fatalities mediate the impact of SPI shocks on out-migration, although the extent of this mediation is relatively small. This result highlights the role of contextual and institutional factors affecting climate-migration linkages (see also Abel et al., 2019) and suggests that droughts give rise to separate channels through per capita GDP and conflicts. One possible explanation for this is that climate shocks can increase social risks within affected populations. This is consistent with other research on the relationship between climate shocks and conflicts (see Xu et al., 2016; Mach et al., 2019). Additional research is necessary to validate this interpretation.

Taken together, our results suggest that more frequent droughts can be expected to increase out-migration in rural areas, both by affecting economy-wide activities and through conflicts. Making local economies more resilient to rainfall shocks, through adaptation strategies or economic transfers, might help reduce the increased variability in rainfall expected with future climate change. The design of such policies could further benefit from a better understanding of destination choices in relation to rural out-migration, which could potentially hasten urbanization, lead to rural-rural displacements, or induce international displacements. In our context, the data was limited with respect to the number of urban provinces, point of destination for migrants, and time-varying irrigation data. This suggests that more evidence on these issues is warranted, and developing our understanding of these migration patterns remains an important research endeavor.

## Figures and Tables

**Fig. 3:**
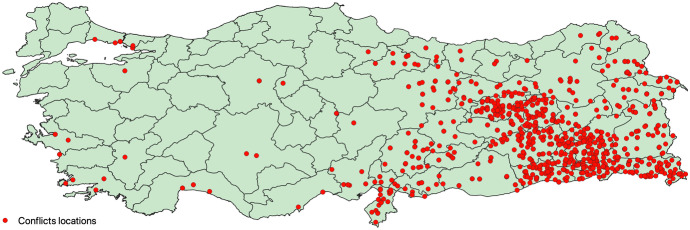
Time trend of conflict fatalities for Turkish provinces

**Fig. 4:**
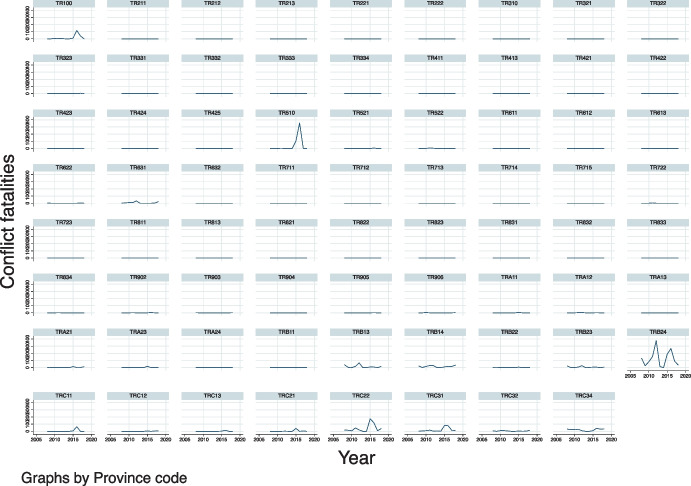
Location of conflict for Turkish provinces

**Fig. 5:**
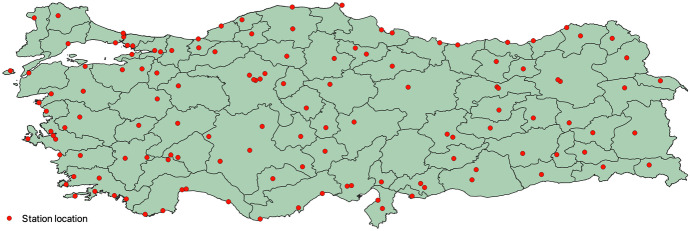
Location of measuring stations for Turkish provinces

**Fig. 6:**
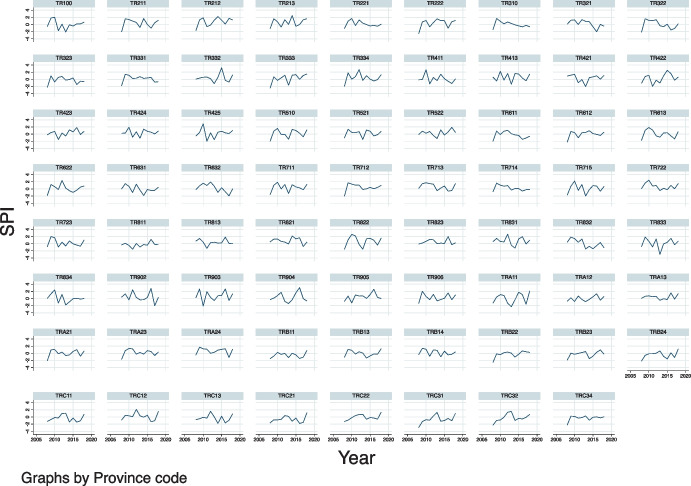
Time trend of SPI for Turkish provinces

## Appendix A. Conflicts

See Figs. 3 and 4

## Appendix B. Location of measuring stations

See Fig. 5

## Appendix C. Rural–urban classification

See Table 7

**Table 7:** List of variables used in Oğdül (2010) six-factors analysis

Categories	Variables
Agricultural production	Percentage of agricultural production in percentage of total agricultural production
	Agricultural production values per 1000 rural residents
	Agricultural production values per 1000 people engaged in agriculture
Non-agricultural production	Level of non-agricultural production
	Employment in industrial sector per total employment
	Employment in construction sector per total employment
	Employment in commercial sector per total employment
	Employment in transportation sector per total employment
	Employment in finance sector per total employment
Employment structure	Employee per total employment
	Women employee per total employment
	Employers per total employment
	Dependency ratio
Demography	Population size
	Rate of urbanization
	Population density
Educational level	Literate per total population
	Literate women per total women population
	Higher education graduates per total population
	Service zone grade for civil servants in education and academics
Trade opportunities	Accessibility (availability of airports, ports and railways)
	Budget income per capita
	Number of branch banks

## Footnotes

1 An empirical association between extreme weather events and agricultural production is also documented in Jayachandran (2006), Iizumi and Ramankutty (2015), Carlo and Bateman (2015), Ochieng et al. (2016), among others.

2 While our data include international out-migration, we cannot use it to quantify separately the effects on national and international out-migration.

3 Note that our baseline analysis uses a 12-month SPI so as to capture yearly rainfall shocks over the growing season. However, we recognize that Türkiye includes several climatic regions with different agricultural systems, and below we discuss the robustness of our results to alternative measures of rainfall shocks.

4 Note that we focus on the total effect of the SPI on out-migration and we do not include control variable into our main estimations (Berlemann & Steinhardt, 2017; Cattaneo & Peri, 2016; Angrist & Pischke, 2009). Nevertheless, in our robustness section, we show that our results remain largely unaffected by the inclusion of key socio-demographic factors variables (education, sex ratio, and population density). We come back to this below.

5 As a robustness check, we also consider alternative measures to identify rural areas (e.g., the share of population working in agriculture). We come back to this below.

6 More formally, the mediated-moderator approach combines a mediation analysis, which identifies a causal sequence between two variables and an outcome, and a moderator analysis, which identifies the effect of a variable on the relationship between another variable and an outcome. See, for example, Muller et al. (2005), Morgan-Lopez and MacKinnon (2006), MacKinnon et al. (2007) for a comprehensive coverage.

7 Benonnier et al. (2019) provide evidence that access to irrigation moderates the temperature-migration relationship, as it shelters yields from weather shocks. We consider this possibility in our set of robustness checks.

8 Bilateral migration data is also used in the literature, see for example Beine and Parsons (2015), Dallmann and Millock (2017) and Abel et al. (2019). However, such data are not available at the provincial level in Türkiye, so we rely on out-migration to identify the role of climate as a push factor in migrations decisions. For other analysis of out-migration data, see for example Feng et al. (2010), Neumann et al. (2015), Nawrotzki et al. (2016), or Debnath and Nayak (2020).

9 We use a total of 130 stations in Türkiye. Appendix B reports their location.

10 In line with this, we measure out-migration in absolute value rather than as a share of total population, since population itself may be affected by climate shocks. In addition our analysis focuses on a 10-year period, so that population size is largely accounted for by the use of province fixed effects, whereas country-wide population growth is captured by time fixed effects. Nevertheless, in the robustness analysis we check whether our estimates remain similar when we scale the outcome variable by provincial population and we add data on the composition of population in each province.

11 We restrict the analysis to 71 provinces with complete precipitation data for at least 30 years. To calculate the provincial SPI, we employ a standardized procedure using the Standard Precipitation Index Generator software of the National Drought Mitigation Center at the University of Nebraska.

12 A 12-month SPI broadly captures agricultural growing seasons (see also Unal et al., 2003; Deniz et al., 2011), although we do not account for potential differences in growing seasons. We discuss alternative periods to define the SPI in the robustness checks.

13 Note that this specification does not include $\textit{SPI}_{i,t}$ as a stand alone variable and we also do not omit one of the three categories. The implication is that we directly estimate a marginal effect for each type of province rather than estimating differences as compared to the omitted category.

14 We exploit geo-coded data about the location of conflict fatalities to determine the yearly number of fatalities per province. See Appendix A.

15 Due to the limitation of TSI data on irrigated areas, we cannot observe the evolution of irrigation installations over time.

16 Figure 6 in Appendix D displays the SPI per province during the observation period. The graphs does not suggest that province-level SPI features a time trend.

17 The decrease in precision in the estimates is likely due to the presence of small provinces in which a relatively small increase in out-migration can result in large proportional effects. This adds noise and reduces the precision of our estimates.
